# Cultural influences on fidelity components in recovery colleges: a study across 28 countries and territories

**DOI:** 10.1136/gpsych-2024-102010

**Published:** 2025-05-27

**Authors:** Yasuhiro Kotera, Amy Ronaldson, Simran Takhi, Simon Felix, Mariam Namasaba, Simon Lawrence, Vanessa Kellermann, Agnieszka Kapka, Daniel Hayes, Danielle Dunnett, Tesnime Jebara, Michio Murakami, Ioannis Bakolis, Julie Repper, Sara Meddings, Vicky Stergiopoulos, Lisa Brophy, Clara De Ruysscher, Lene Eplov, Charlotte Toernes, Dagmar Narusson, Bernd Puschner, Ramona Hiltensperger, Yuki Miyamoto, Stynke Castelein, Trude Gøril Klevan, Hannah Morland-Jones, Edith Moore, Samson Tse, Michael Ryan, Gianfranco Zuaboni, Charlotte Hanlon, Laura Asher, Wouter Vanderplasschen, Susana Ochoa, Jonna Tolonen, Ashleigh Charles, Mário Andrade, Daniel Elton, Peter Bates, Julie Cooper, Jason Grant, Claire Henderson, Mike Slade

**Affiliations:** 1 Faculty of Medicine and Health Sciences, University of Nottingham, Nottingham, UK; 2 Center for Infectious Disease Education and Research, The University of Osaka, Suita, Japan; 3 Health Service and Population Research Department, King's College London, Institute of Psychiatry, Psychology and Neuroscience, De Crespigny Park, London, UK; 4 Department of Psychology, University of Bordeaux, Bordeaux, France; 5 Research Department of Behavioural Science and Health, Institute of Epidemiology & Health Care, University College London, London, UK; 6 ImROC, Nottingham, UK; 7 Department of Psychiatry, University of Toronto, Toronto, Ontario, Canada; 8 School of Allied Health, Human Services and Sport, College of Science, Health and Engineering, La Trobe University, Melbourne, Victoria, Australia; 9 Centre for Mental Health, Melbourne School of Population and Global Health, The University of Melbourne, Melbourne, Victoria, Australia; 10 Department of Special Needs Education, Ghent University, Ghent, Belgium; 11 EQUALITY Research Collective, University of Applied Sciences and Arts, Ghent, Belgium; 12 Copenhagen Research Unit for Recovery, Mental Health Centre Amager, Copenhagen, Denmark; 13 University of Tartu, Institute of Social Studies, Tartu, Estonia; 14 Department of Psychiatry II, Ulm University, Günzburg, Germany; 15 Department of Psychiatric Nursing, Graduate School of Medicine, The University of Tokyo, Bunkyo-ku, Japan; 16 Faculty of Behavioural and Social Sciences, Department of Clinical Psychology and Experimental Psychopathology, Lentis Psychiatric Institute, Lentis Research, University of Groningen, Groningen, The Netherlands; 17 Department of Health, Social and Welfare Studies, University of South-Eastern Norway, Kongsberg, Norway; 18 Division of Mental Health Services, Akershus University Hospital, Lørenskog, Norway; 19 Cardiff and Vale Recovery & Wellbeing College, Whitchurch, UK; 20 Drive Direction, Manukau, New Zealand; 21 Department of Social Work and Social Administration, The University of Hong Kong, Hong Kong, China; 22 Community Health Organisation Health Service Executive (HSE), Dublin, Ireland; 23 Recovery College Berne, University Hospital of Psychiatry and Psychotherapy, University Berne Psychiatric Services, Berne, Switzerland; 24 Division of Psychiatry, Centre for Clinical Brain Sciences, University of Edinburgh, Edinburgh, UK; 25 Department of Psychiatry, School of Medicine, College of Health Sciences, Addis Ababa University, Addis Ababa, Ethiopia; 26 MERITT Group, Institut de Recerca Sant Joan de Deu, Parc Sanitari Sant Joan de Deu, CIBERSAM, ISCIII, Sant Boi de Llobregat, Spain; 27 Unit of Population Health, University of Oulu, Oulu, Finland; 28 Department of Psychology, Federal University of São João del-Rei, São João del-Rei, Brazil; 29 RECOLLECT Lived Experience Advisory Panel, London, UK; 30 Faculty of Nursing and Health Sciences, Health and Community Participation Division, Nord University, Namsos, Norway

**Keywords:** mental health services, ethnopsychology, adaptation, psychological, mental health

## Abstract

**Background:**

Recovery colleges (RCs) support personal recovery through education, skill development and social support for people with mental health problems, carers and staff. Guided by co-production and adult learning principles, RCs represent a recent mental health innovation. Since the first RC opened in England in 2009, RCs have expanded to 28 countries and territories. However, most RC research has been conducted in Western countries with similar cultural characteristics, limiting understanding of how RCs can be culturally adapted. The 12-item Recovery Colleges Characterisation and Testing (RECOLLECT) Fidelity Measure (RFM) evaluates the operational fidelity of RCs based on 12 components, but cultural influences on these components remain underexplored.

**Aims:**

To assess associations between Hofstede’s cultural dimensions and RFM items to identify cultural influences on fidelity components.

**Methods:**

A cross-sectional survey of RC managers was conducted across all 221 RCs. Mixed-effects regression models examined associations between Hofstede’s country-level cultural dimensions and item-level RFM scores, adjusted for healthcare expenditure and income inequality. Four cultural dimensions, obtained from Hofstede, were analysed: individualism (prioritising personal needs), indulgence (enjoyment-oriented), uncertainty avoidance (preference for predictability) and long-term orientation (future-focused).

**Results:**

The RFM was completed by 169 (76%) RC managers. Seven RFM items showed associations with cultural dimensions. Equality was linked to short-term orientation, while learning was associated with individualism and uncertainty avoidance. Both individualism and indulgence influenced co-production and community focus. Commitment to recovery was shaped by all four cultural dimensions, with the strongest associations seen for individualism and indulgence. Individualism enhanced explicit focus on strengths-based practice, while uncertainty avoidance influenced course distinctiveness.

**Conclusions:**

This study demonstrates how culture shapes RC fidelity components, providing actionable insights for cultural adaptation. Incorporating under-represented dimensions, such as collectivism and restraint, could improve the RFM’s global applicability, facilitating implementation. Future research should explore cultural nuances, engage diverse stakeholders and refine fidelity measures to enhance RC inclusivity and effectiveness worldwide.

**WHAT IS ALREADY KNOWN ON THIS TOPIC**

- Recovery colleges (RCs) promote personal recovery through co-production and adult learning, with a global presence across 28 countries and territories, although most research has focused on Western contexts.
- The 12-item RECOLLECT Fidelity Measure (RFM), rated by RC managers, assesses key operational components, although specific guidance for cultural adaptation remains unknown.

**WHAT THIS STUDY ADDS**

- This first global study identified that seven of 12 RC operational components are influenced by cultural characteristics—most notably individualism—highlighting both the under-representation of collectivistic values and opportunities to enhance cultural inclusivity in current RC models.
- These findings provide critical insights for the global cultural adaptation of RCs, such as integrating collectivistic values (eg, prioritising group harmony in co-production) and restraint values (eg, balancing self-control with individual expression in learning processes) into RC operations and tools.

**HOW THIS STUDY MIGHT AFFECT RESEARCH, PRACTICE OR POLICY**

- This study can affect research by informing refinement of the RFM to reflect cultural influences, guiding practice through culturally inclusive operations and training focused on key components and supporting policy by promoting culturally responsive implementation and scale-up of RCs globally.

## Introduction

 Recovery colleges (RCs) are a relatively new mental health recovery support approach focused on education, skill development and social support. RCs are widely regarded as a mental health innovation and are designed for people with mental health problems, their informal carers and mental health staff.^1^ RCs trace their roots to the peer-run recovery education centres that emerged in the USA during the 1990s. The first RC opened in England in 2009, and the model has since spread to 28 countries and territories across Europe, Asia, Africa, North America and Oceania.^2^ RCs operate in diverse settings, including primary and secondary healthcare, non-governmental organisations and educational institutions.

Two key principles underpin RCs: co-production and adult education.^1^ Co-production involves integrating the expertise of people with lived experience of mental health problems and professionals in the planning, delivery and evaluation of courses. Adult education refers to self-directed learning, where people engage in strengths-based, person-centred, inclusive and community-focused education. Together, these principles support personal recovery, enabling individuals to live fulfilling and autonomous lives despite mental health challenges. Additionally, RCs foster social inclusion by empowering students to take on social and economic roles.^3^ Courses cover topics such as recovery planning, understanding mental health problems and diagnoses, life skills and pathways to becoming peer trainers. These initiatives aim to enhance self-confidence, quality of life and social engagement among students.

To understand how RCs work, a change model for service user students was developed through an iterative process combining analysis of key publications and stakeholder validation.^4^ The change model identifies mechanisms of action and outcomes. The mechanisms of action explain how RCs function, including (a) creating an empowering environment, (b) facilitating new types of relationships, (c) supporting personal growth and (d) reducing power differentials through co-production. The outcomes demonstrate the impact of RCs, showing changes in students themselves, such as improved self-confidence and self-management, as well as in their lives, including greater social engagement and development of personal interests.

### Evidence of effectiveness and cost-effectiveness

Evidence of effectiveness and cost-effectiveness of RCs is still emerging, with methodological limitations noted in existing studies.^4^,^7^ However, early findings suggest positive outcomes for students and staff. Students report improved self-esteem, hope, quality of life and reduced stigma.^6^ Staff benefit from enhanced skills, more positive attitudes towards co-production and increased motivation.^5^ Preliminary evidence also points to cost-effectiveness. For example, one study found that students who attended RCs used fewer healthcare services over 18 months, including reduced hospitalisations.^8^ In Australia, a cost-benefit analysis estimated net savings of $A269 per student due to decreased emergency and inpatient service use.^9^

A key contributor to the underdeveloped evidence base for RCs is the lack of standardisation in their operations.^10^ To address this, the Recovery Colleges Characterisation and Testing (RECOLLECT) Fidelity Measure (hereafter ‘RFM’) was developed through a systematised review, expert consultations and stakeholder interviews (online supplemental material 1).^10^ The RFM is grounded in 12 key operational components of RCs, which were categorised into seven non-modifiable components (essential, universal elements such as equality and co-production) and five modifiable components (adaptable elements, such as distinctiveness of course content, to suit specific contexts). Each component is assessed via a single item, with the 12-item measure rated by RC managers. The total fidelity score, calculated as the sum of the seven non-modifiable components, ranges from 0 (low fidelity) to 14, with higher scores indicating closer alignment with key operational components deemed essential for RC operation. While the RFM has been used internationally to evaluate RC fidelity, its cultural adaptation and relevance across diverse settings remain unexamined, presenting a critical gap for future research.

### Cross-cultural considerations

Most RC research has been conducted in Western, Educated, Industrialised, Rich and Democratic (WEIRD) countries, which represent only 12% of the global population.^11^ To date, six reviews on RCs have been published, and all the included studies (n=185) were conducted in WEIRD countries.^4^,^610 12 13^ The limited evidence from non-WEIRD countries highlights the need for cross-cultural studies to understand how RCs function in diverse cultural contexts. Recently, a discourse analysis study comparing RC implementation in England and Japan was conducted and found different emphases in the advertisement texts (eg, ‘self-management’ in England vs ‘learning together’ in Japan).^14^ Moreover, cross-cultural influences such as self-enhancement and in-group biases are emerging as impacting fidelity.^15^ Overall, evidence on RCs from non-WEIRD countries remains scarce, and fostering cross-cultural understanding of RCs could help bridge this knowledge gap.

Cultural adaptation is essential for implementing RCs across diverse contexts, particularly in non-WEIRD settings, as cultural values significantly shape mental health experiences.^16 17^ Evidence shows that culturally adapted treatments are more effective, with greater symptom remission rates and improved mental health outcomes.^18^ This highlights the importance of evaluating how RCs can be tailored to different cultural contexts to maximise their impact. However, what cultural characteristics may influence the assessment of fidelity in relation to specific components of RC operation remains unknown.

Hofstede’s cultural dimension theory is the most widely used quantitative framework in cross-cultural research. It defines culture as ‘the collective programming of the mind that distinguishes the members of one group or category of people from others’.^19^ The theory identifies six cultural characteristics: individualism, indulgence, uncertainty avoidance, long-term orientation, power distance and success-drivenness (table 1).

**Table 1:** Six cultural characteristics in the cultural dimension theory

Characteristic (interpretation)	Meaning
Individualism (vs collectivism)	The degree to which a society expects individuals to be loosely tied to one another and to take care of only themselves and their immediate family.
Indulgence (vs self-restrained)	Acceptance of relatively free gratification of basic and natural human needs to enjoy life.
Uncertainty avoidance (high vs low)	The degree to which individuals feel threatened by unknown situations and try to avoid such situations.
Long-term orientation (vs short-term orientation)	Values oriented towards future rewards, perseverance and thrift, which are related to ‘saving’ as opposed to ‘spending’.
Power distance (high vs low)	The degree to which inequality and unequal distributions of power between parties are accepted.
Success-drivenness (vs quality-orientation)	The societal value of achievement and material rewards for success.

Our previous study identified that RCs in countries oriented to individualism, indulgence, uncertainty acceptance (ie, low uncertainty avoidance) and short-term orientation (ie, low long-term orientation) tended to have higher total fidelity scores on the RFM.^11^ These four cultural characteristics are generally more prominent in WEIRD countries. This formative study empirically supported the presence of cultural influence in the fidelity of RC operations. However, the relationship between individual items of the RFM and cultural characteristics has not been investigated. Without this understanding, RC staff lack clear guidance on which aspects of their operations may need to be adapted and how to implement these changes effectively.

### Study aim

This study aimed to identify associations between Hofstede’s cultural dimension indices and each item of RFM in all RCs currently operating around the world.

## Methods

### Design

We conducted a cross-sectional, observational survey in two phases: first, surveying all RCs in England (‘England survey’),^20^ followed by RCs in other countries and territories (‘international survey’).^2^

All RCs, whose managers completed the RFM from August to October 2021 for the England survey, and from February to October 2022 for the international survey, were included. This study is a post hoc analysis of data obtained from both England^20^ and international surveys,^2^ focusing on the cultural aspects of RCs and informed by a previous hypothesis-generating cross-cultural analysis of the same dataset.^11 14 15 21^ The Strengthening the Reporting of Observational Studies in Epidemiology guidelines were followed (online supplemental material 2).

### Procedures

Three steps were undertaken for both surveys: (1) establishing RC inclusion criteria, (2) identifying and contacting eligible RCs and (3) distributing and collecting the survey. These steps were led by DH.

#### Establishing RC inclusion criteria

Since not all RCs are identified as a ‘recovery college’ (eg, ‘recovery academy’), we included any services that met three criteria, based on key RC components^10^: (a) a focus on supporting personal recovery, (b) an emphasis on co-production and (c) use of adult learning principles, all confirmed by service managers. Full details are reported elsewhere.^2^

#### Identifying and contacting eligible RCs

For the England survey, four methods were used to identify potentially eligible RCs in June and July 2021: (a) online searches, (b) consultation with national RC leaders and recovery networks, including ImROC (imroc.org), (c) snowball sampling and (d) phone calls to host charities and mental health service providers. The research team then contacted the identified services to confirm their eligibility based on the inclusion criteria.

For the international survey, we first identified countries and territories with operating RCs. An initial list was made through (a) an RC international survey,^22^ (b) inquiries to existing RC organisations, (c) consultations with 23 recovery experts and (d) communications with collaborators in countries and territories offering similar services (eg, peer support). Next, we identified country leads in these listed countries and territories through networks developed in the initial phase. Each country lead conducted a local-language literature search to locate RCs in their country. Finally, country leads consulted with service managers to confirm eligibility based on inclusion criteria and used snowball sampling, where managers identified additional eligible services.

#### Distributing and collecting the survey

For the England survey, a pilot was developed using the Checklist for Reporting Results of Internet E-Surveys guidelines,^23^ revised based on expert feedback and completed by two RC managers. Eligible service managers were asked to complete the final survey, including the RFM on Qualtrics, an online survey platform.

The international survey was based on the England survey, with adjustments made to specific phrases (eg, ‘NHS services’ to ‘health services’). It was piloted by three RC experts from Australia, Canada and Japan. The final version, including the RFM, was distributed to RC managers by country leads, using either Qualtrics or Microsoft Word formats. In non-English-speaking countries and territories with multiple RCs, the survey was translated by the country leads and checked by a second translator. Seven language versions were created (Danish, Dutch, French, German, Japanese, Mandarin-Chinese and Norwegian).^2^ Completed Qualtrics responses were directly accessible to the research team, while Microsoft Word responses were encrypted and emailed by RC managers or country leads, then entered into Qualtrics. Data from both surveys were integrated. No financial incentives were offered in either survey.

### Eligible RCs

For the England survey, 134 services were initially identified, with 88 (66%) confirmed as eligible. Forty-six services were excluded, mainly due to being non-contactable or no longer in operation (n=20).

**Figure 1:**
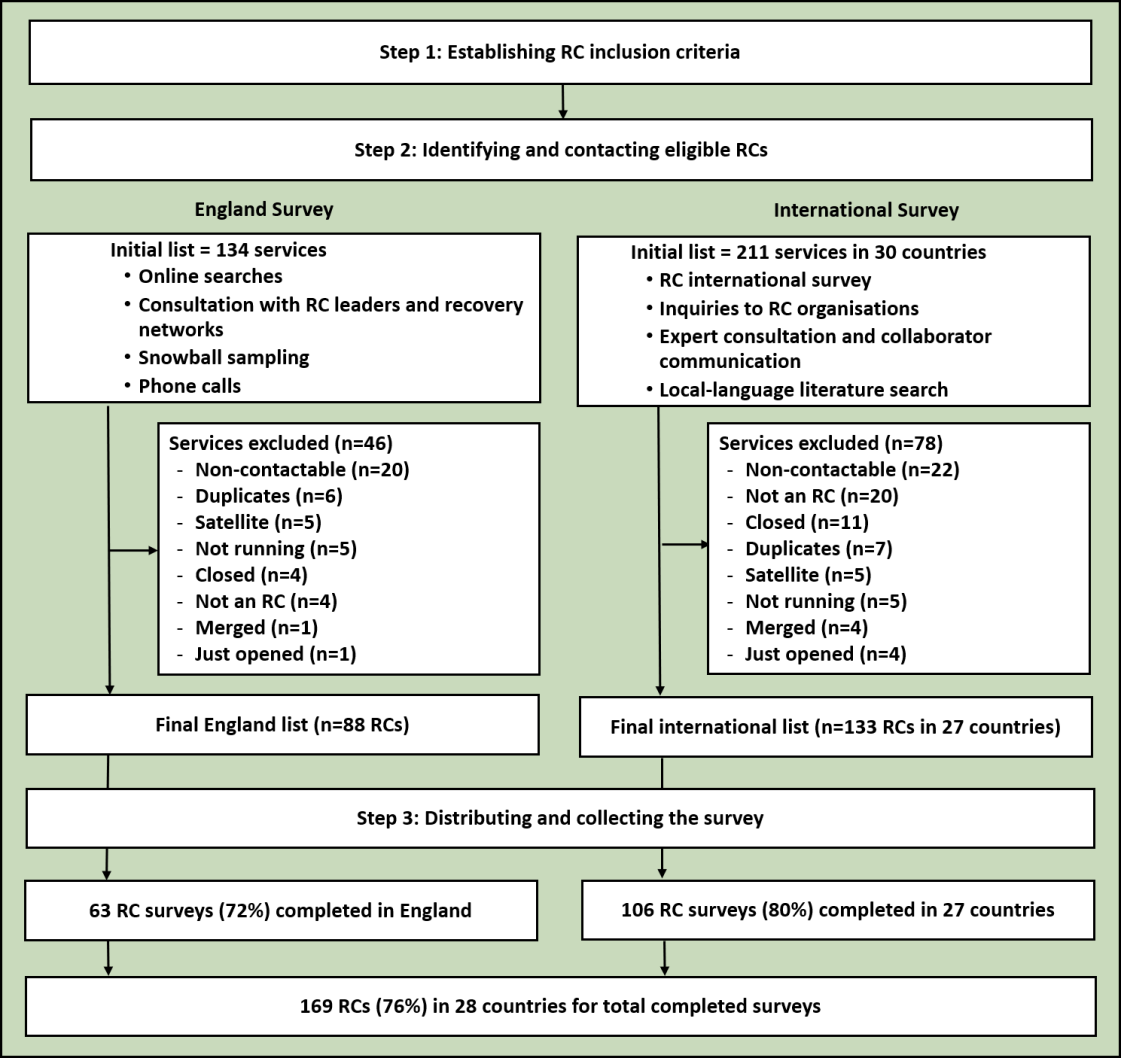
Summary of the flowchart of RC participation. RC, recovery college.

For the international survey, 49 countries and territories were initially listed. After expert consultation and searches by country leads, the final list included 30 countries and territories with 211 potential RCs. Two countries and territories and 78 RCs were excluded for not meeting the inclusion criteria, primarily due to being non-contactable or no longer operating (n=22). Figure 1 illustrates the flowchart of RC participation.

### Measures

The RFM,^10^ rated by RC managers, assessed the item-level scores on fidelity (non-modifiable items) and operational types (modifiable items), which were entered as outcome variables. Each item assesses one component of RC operation. Seven items evaluate non-modifiable components, and five items evaluate modifiable components of RC operation. The seven non-modifiable components are: (1) equality, (2) adult learning, (3) tailoring to the student, (4) co-production, (5) social connectedness, (6) community focus and (7) commitment to recovery, responded on a 3-point ordinal scale from 0 (low fidelity) to 2.^10^ The five modifiable components are: (8) available to all, (9) location, (10) distinctiveness of course content, (11) strengths-based and (12) progressive, each assessed using a categorical variable with either type 1 or type 2 responses. The modifiable components can only inform the types, not high or low fidelity. Table 2 presents a summarised version of the RFM (for the full measure, please see online supplemental material 1). The measure satisfies scaling assumptions, demonstrating adequate internal consistency (0.72), test–retest reliability (0.60), content validity and discriminant validity.^10^

**Table 2:** Summarised version of the RECOLLECT Fidelity Measure

Non-modifiable components	0 (low fidelity) to 2
Equality	0–2
Adult learning	0–2
Tailoring to the student	0–2
Co-production	0–2
Social connectedness	0–2
Community focus	0–2
Commitment to recovery	0–2
Total fidelity score (sum of 1–7)	0*–*14

Modifiable components	Type 1	Type 2
Available to all	The recovery college is available to all.	The recovery college is limited to specific groups.
Location	The recovery college is based in a community location that is not shared with health, social care or other statutory services.	The recovery college is based in a location which is shared with health, social care or other statutory services.
Distinctiveness of course content	Any topic can be offered as a course, irrespective of whether it is available in mainstream adult education settings.	Only topics not available in mainstream adult education settings are offered.
Strengths-based	A focus on strengths (not problems) is implicit in the college.	A focus on strengths (not problems) is explicit in the college, in addition to dimensions 1–7 above.
Progressive	There is a focus on ‘being’ and ‘belonging’, not on goal setting.	There is a focus on ‘becoming’ and a strong emphasis on goal setting and change.

RECOLLECT, Recovery Colleges Characterisation and Testing.

The Value Survey Module 2013, sourced from Hofstede,^24^ was used to assess cultural characteristic data, which were entered as predictor variables. This self-report measure comprises 24 items rated on a 5-point Likert scale (1–5).^24^ For example, it asks how important ‘keeping time free for fun’ is, rated from 1 being ‘of utmost importance’ to 5 being ‘of very little or no importance’. Each cultural characteristic score is derived from the mean of four items and index formulas provided in the manual, with scores rank-ordered to range from 0 (low) to 100 based on comparison with other countries and territories. Four cultural characteristics were entered as predictors due to their association with the total scores of the seven non-modifiable RFM items^11^: individualism, indulgence, uncertainty avoidance and long-term orientation.

Two confounding variables were included in the analyses, as they are relevant to mental health treatment resources.^11^ The percentage of gross domestic product (GDP) spent on health represents healthcare spending relative to the size of the economy, calculated by dividing total health expenditure by GDP.^25^ The Gini coefficient for each country and territory reflects income inequality, ranging from 0 (perfect equality) to 1 (maximum inequality), and was obtained from the World Bank.^26^

### Statistical analysis

Statistical analysis was led by AR. Item-level fidelity scores were summarised as medians and interquartile ranges (IQRs) for countries and territories with fidelity data from multiple RCs. To examine adjusted associations between the four country-level cultural characteristics and each of the seven college-level non-modifiable fidelity items, we used mixed-effects ordinal logistic regression models with a country-level random intercept to account for between-country variability, with results reported for 10-unit changes in the cultural scores to facilitate interpretation. Associations between each cultural characteristic and the modifiable fidelity items (type 1 vs type 2) were assessed using adjusted mixed-effects logistic regressions. Adjusted models included the percentage of GDP spent on healthcare and the Gini coefficient for each country and territory as potential confounders. Power calculation was not required for this exploratory study.^27^ Data for individualism and uncertainty avoidance were missing for Uganda, so this country was excluded from analyses involving these cultural predictors. Gini coefficients for China (in Hong Kong) and New Zealand were unavailable from the World Bank (personal communication, 28 April 2023, The World Bank, Development Economics Data Group); these countries and territories were omitted from adjusted mixed-effects linear regression models, but descriptive data were provided. All analyses were performed using STATA V.17.0 (StataCorp, College Station, Texas, USA).

## Results

### Participating RCs

The two surveys identified that in 2021/2022, there were 221 RCs in 28 countries and territories across Europe, Asia, Africa, North America and Oceania. A total of 169 (76%) RC managers from these countries and territories completed the surveys, representing RCs with over 55 000 students attending in total.^2 20^ Jersey (not counted as a country) had one operational RC that responded to the survey; however, it was excluded from the analysis due to the lack of available cultural characteristic data. Sample characteristics are presented in table 3, along with the four Hofstede cultural dimensions identified in previous research as influencing RFM scores.

**Table 3:** Sample characteristics of participating RCs, including response rates, national economic indicators and cultural characteristics by country and territory

Country and territory(n=28)	Recovery college (n=168/220: responded/total)	% GDP spent on healthcare	Gini coefficient	Individualism	Uncertainty avoidance	Long-term orientation	Indulgence
Africa (n=1)	2/2						
Uganda	2/2	3.8	42.8	Not available	Not available	24	52
Asia (n=3)	13/15						
China (in Hong Kong)	2/2	5.3	–	25	29	61	17
Japan	9/11	10.7	32.9	46	92	88	42
Thailand	2/2	3.8	36.4	20	64	32	45
Europe (n=21)	128/170						
Belgium	10/14	10.7	27.2	75	94	82	57
Bulgaria	1/1	7.1	41.3	30	85	69	16
Czechia	1/1	7.8	25.0	58	74	70	29
Denmark	9/9	10.0	28.2	74	23	35	70
United Kingdom (in England)	63/88	10.1	35.1	89	35	51	69
Estonia	2/2	6.7	30.3	60	60	82	16
Finland	2/2	9.1	27.3	63	59	38	57
France	1/1	11.1	32.4	71	86	63	48
Germany	3/3	11.7	31.7	67	65	83	40
Hungary	2/3	6.3	29.6	80	82	58	31
Iceland	1/1	8.6	26.1	60	50	28	67
Ireland	7/11	6.7	30.6	70	35	24	65
Italy	4/4	8.7	35.2	76	75	61	30
The Netherlands	2/2	10.1	28.1	80	53	67	68
United Kingdom (in Northern Ireland)	3/4	10.1	35.1	89	35	51	69
Norway	4/5	10.5	27.6	69	50	35	55
United Kingdom (in Scotland)	3/3	10.1	35.1	89	35	51	69
Spain	3/6	9.1	34.7	51	86	48	44
Sweden	3/3	10.9	30.0	71	29	53	78
Switzerland	3/4	11.3	33.1	68	58	74	66
United Kingdom (in Wales)	1/2	10.1	35.1	89	35	51	69
Oceania (n=2)	9/11						
Australia	7/9	9.9	34.3	90	51	21	71
New Zealand	2/2	9.7	–	79	49	33	75
North America (n=1)	16/23						
Canada	16/23	10.8	33.3	80	19	36	68

GDP, gross domestic product; RC, recovery college.

Most RCs (159; 94%) were located in WEIRD countries, apart from those in China (in Hong Kong), Japan, Thailand and Uganda.

### Associations between culture and fidelity

Fidelity scores for each item per country and territory are presented in online supplemental material 3. Adjusted associations between the four cultural characteristics and the fidelity measure items are presented in table 4.

**Table 4:** Adjusted associations between cultural characteristics and scores on fidelity items (mixed-effects logistic and ordinal logistic regression)

	Non-modifiable fidelity items (ordinal logistic regression)						
	Equality	Learning	Tailored to students	Co-production	Social connectedness	Community focus	Commitment to recovery
	IRR (95% CI)	IRR (95% CI)	IRR (95% CI)	IRR (95% CI)	IRR (95% CI)	IRR (95% CI)	IRR (95% CI)
Individualism (n=163)	1.02(0.67 to 1.54)	1.54(1.19 to 1.98)*	1.50(0.93 to 2.43)	1.69(1.31 to 2.19)**	0.99(0.78 to 1.25)	1.37(1.09 to 1.72)*	1.42(1.11 to 1.80)*
Indulgence (n=165)	1.38(0.97 to 1.98)	1.27(0.91 to 1.77)	0.94(0.58 to 1.54)	1.36(1.02 to 1.83)*	0.96(0.75 to 1.24)	1.41(1.09 to 1.81)*	1.58(1.21 to 2.07)*
Uncertainty avoidance (n=163)	0.81(0.64 to 1.02)	0.82(0.70 to 0.96)*	0.87(0.62 to 1.22)	0.86(0.69 to 1.06)	0.92(0.80 to 1.07)	0.87(0.75 to 1.00)	0.77(0.66 to 0.90)*
Long-term orientation (n=165)	0.75(0.61 to 0.92)*	0.83(0.66 to 1.05)	0.94(0.65 to 1.36)	0.80(0.64 to 1.00)	1.03(0.86 to 1.23)	0.88(0.71 to 1.09)	0.75(0.61 to 0.92)*

	Modifiable fidelity items (logistic regression)				
	Available to all	Location	Distinctiveness of course content	Strengths-based	Progressive
	OR (95% CI)	OR (95% CI)	OR (95% CI)	OR (95% CI)	OR (95% CI)
Individualism (n=163)	1.09(0.82 to 1.45)	1.08(0.84 to 1.37)	1.23(0.96 to 1.58)	1.64(1.24 to 2.16)**	1.07(0.81 to 1.40)
Indulgence (n=165)	1.10(0.82 to 1.48)	1.15(0.88 to 1.50)	1.16(0.84 to 1.62)	1.30(0.91 to 1.87)	0.94(0.71 to 1.25)
Uncertainty avoidance (n=163)	0.91(0.76 to 1.09)	0.92(0.79 to 1.07)	0.83(0.71 to 0.98)*	0.83(0.64 to 1.07)	0.97(082 to 1.16)
Long-term orientation (n=165)	0.90(0.73 to 1.12)	0.88(0.73 to 1.07)	0.91(0.71 to 1.16)	0.93(0.71 to 1.22)	1.02(0.83 to 1.26)

IRRs and ORs are reported for 10-unit changes in the cultural scores to facilitate interpretation. The numbers in the second row correspond to those in the RECOLLECT Fidelity Measure. Covariates: % GDP spent on healthcare, Gini coefficient. Predictor variables=cultural characteristics (individualism, indulgence, uncertainty avoidance and long-term orientation). Outcome variables=fidelity items.

* p<0.05, **p<0.001.

CI, confidence interval; GDP, gross domestic product; IRR, incidence rate ratio; OR, odds ratio; RECOLLECT, Recovery Colleges Characterisation and Testing.

### Non-modifiable items

Among the seven non-modifiable fidelity items, five showed significant associations with at least one of the four cultural characteristics examined.

#### Equality

Countries and territories with a short-term orientation (rather than long-term orientation) demonstrated higher scores on equality at the RC level (incidence rate ratio (IRR) 0.75, 95% confidence interval (CI) 0.61 to 0.92, p=0.005). This finding suggests that RCs in cultures with a short-term orientation are more likely to emphasise equality.

#### Learning

Higher levels of individualism (IRR 1.54, 95% CI 1.19 to 1.98, p=0.001) and uncertainty acceptance (as opposed to uncertainty avoidance) (IRR 0.82, 95% CI 0.70 to 0.96, p=0.012) were associated with higher scores on learning. These results imply that RCs in countries and territories valuing individualism and uncertainty acceptance may place a stronger emphasis on learning.

#### Co-production and community focus

Both individualism and indulgence were positively associated with co-production (individualism: IRR 1.69, 95% CI 1.31 to 2.19, p<0.001; indulgence: IRR 1.36, 95% CI 1.02 to 1.83, p=0.039) and community focus (individualism: IRR 1.37, 95% CI 1.09 to 1.72, p=0.007; indulgence: IRR 1.41, 95% CI 1.09 to 1.81, p=0.008). These findings indicate that RCs in more individualistic and indulgent cultures are more likely to prioritise co-production and community engagement.

#### Commitment to recovery

All four cultural characteristics—individualism (IRR 1.42, 95% CI 1.11 to 1.80, p=0.004), indulgence (IRR 1.58, 95% CI 1.21 to 2.07, p=0.001), uncertainty acceptance (IRR 0.77, 95% CI 0.66 to 0.90, p=0.001) and short-term orientation (IRR 0.75, 95% CI 0.61 to 0.92, p=0.005)—were significantly associated with higher scores on commitment to recovery. This underscores the importance of cultural characteristics in shaping RCs’ focus on recovery-oriented practices.

### Modifiable items

Among the five modifiable fidelity items, two were associated.

#### Strengths-based

Higher levels of individualism were associated with type 2 in strengths-based fidelity, reflecting a stronger emphasis on an explicit focus on strengths (OR 1.64, 95% CI 1.24 to 2.16, p<0.001).

#### Distinctiveness of course content

Higher levels of uncertainty avoidance were linked to type 1 in distinctiveness of course content, which emphasises the provision of both mainstream and recovery-specific content (OR 0.83, 95% CI 0.71 to 0.98, p=0.027).

## Discussion

This study investigated associations between Hofstede’s cultural dimensions and each item of the RFM in all RCs operating in the world. The results revealed notable links between cultural characteristics and both non-modifiable and modifiable fidelity items. Of the 12 items, seven were associated with at least one cultural characteristic. These findings underscore the substantial cultural influences shaping RC operations.

### Main findings

The association between short-term orientation and higher equality scores reflects the emphasis on practical and egalitarian approaches in short-term-oriented cultures.^19^ No previous studies have identified this relationship. The RFM’s description of equality highlights valuing contributions regardless of background or mental health status.^10^ Short-term orientation values immediate, tangible outcomes,^19^ aligning with the drive to foster inclusive and balanced interactions within RCs. By avoiding hierarchical structures, short-term-oriented cultures may further enable equal contributions from students and trainers. Conversely, long-term-oriented cultures may prioritise gradual, future-focused deliberations, incorporating sufficient reflection to identify unintended or unwanted consequences before they arise. This could limit the immediacy of equality-driven outcomes in RC settings.

The positive association between individualism and learning is consistent with the description of learning as fostering autonomy, responsibility and reflective exercises.^10^ This reflects the emphasis placed on autonomous learning in many individualistic countries.^28 29^ Individualistic cultures emphasise self-directed learning, personal growth and self-management, which align with the adult education principles described in the RFM.^14^ RCs in countries and territories oriented to individualism may be more accustomed to and accepting of autonomous learning and self-management. Similarly, the link between uncertainty acceptance and learning suggests that RCs in countries and territories oriented to uncertainty acceptance tolerate ambiguity and exploration and are more inclined to adopt interactive and reflective learning approaches, compared with RCs in countries and territories oriented to uncertainty avoidance. This cultural openness fosters the collaborative and exploratory learning environments emphasised in RCs.

Co-production and community focus items were influenced by individualism and indulgence. The co-production item in the RFM emphasises collaboration between people with lived experience and professionals. However, in collectivistic cultures, where group harmony is prioritised,^19^ the blurring of roles inherent in co-production may be perceived as disruptive. Indeed, the blurring of roles was identified as a challenge in collectivistic organisations.^30^,^32^ Similarly, restraint (ie, low indulgence) emphasises self-control and group harmony over self-expression,^19^ which may conflict with the RFM’s emphasis on amplifying individual voices, as such expressions could be seen as indulgent. Community focus, which highlights the integration of RCs within local communities, is also shaped by individualism and indulgence. While the RFM description stresses the importance of relationships with others, the assessment of these relationships is made by RC managers. In collectivistic and restraint-oriented cultures, this approach may be challenging, as relationships are often judged collectively rather than individually.^33 34^ For these cultures, understanding the nature of relationships may require input from all parties involved, reflecting fundamental differences in how relationships are perceived and evaluated across cultural contexts.

RCs in countries and territories with high levels of individualism, indulgence, uncertainty acceptance and short-term orientation exhibited greater commitment to recovery. The description of the commitment to recovery item emphasises the positive energy of RC staff towards students’ recovery.^10^ In individualistic cultures, RC managers may prioritise fostering student empowerment and autonomy, aligning with the recovery-oriented principles of shared values and dedication. Indulgent cultures, with their emphasis on well-being and enjoyment, may encourage managers to create vibrant and supportive recovery environments. Uncertainty acceptance may foster adaptability and openness to innovative practices, allowing RC managers to explore creative ways of actively supporting recovery. Lastly, short-term orientation may drive a focus on visible, actionable recovery outcomes, ensuring immediate progress. Together, these cultural characteristics likely contribute to RCs cultivating an environment defined by optimism, dedication and a strong focus on recovery principles.

For the five modifiable items, RCs in individualistic countries and territories were more likely to explicitly focus on strengths-based practices, whereas RCs in collectivistic countries and territories reported a more implicit approach. This difference may be attributed to the self-enhancement tendencies of individualistic cultures, where fostering and expressing a highly positive self-image is encouraged and accepted.^15^ In RCs influenced by individualism, explicitly highlighting strengths-based practices aligns with their cultural norms. In contrast, self-enhancement is less accepted in collectivistic cultures, where self-effacement is more valued—particularly in East Asia,^33^ which includes two of the three participating Asian countries and territories. For people oriented to collectivism, the term ‘strengths’ might carry negatively valorised connotations of self-promotion and could be perceived as a threat to group harmony. Instead, terms such as ‘values’, which emphasise internal personal significance rather than external comparison, may be more culturally acceptable, as they help avoid potential conflicts with the collectivist emphasis on group harmony.

RCs in uncertainty-avoidant cultures reported a wider offer of mainstream courses. This aligns with the ‘just-in-case’ mindset of uncertainty-avoidant cultures, which prefer to minimise uncertainty by adhering to tradition and norms.^19^ Notably, universities in uncertainty-avoidant cultures face great pressure to offer traditional courses.^35^ As RCs are a relatively new initiative, RCs in countries and territories oriented to uncertainty avoidance may feel hesitant to focus exclusively on non-mainstream courses, as these might be perceived as risky or unconventional. Offering both mainstream and non-mainstream courses allows RCs to balance tradition with innovation, accommodating diverse preferences while reducing uncertainty.

The novelty of this global cross-cultural study, involving RCs across 28 countries and territories, lies in identifying specific components of RC operations that require focus during cultural adaptation. These findings provide actionable insights into how RCs can be tailored to different cultural contexts. While previous studies have highlighted the importance of cultural adaptation for RCs—especially when implemented in non-WEIRD countries^2 11^—they have not provided specific, actionable guidance on operational priorities. Service disparities affecting minority cultures remain a pressing issue both globally (eg, marginalised Indigenous populations) and within individual countries (eg, people in ethnic minority groups), linked to inequities in service uptake, poorer mental health outcomes and increased healthcare costs.^36^ RCs are currently active in 28 countries and territories, including two low- and middle-income countries and territories. This study identifies priority areas for cultural adaptation, facilitating the development of culturally competent RCs.

Additionally, many countries and territories still only have a few RCs (eg, 13 countries and territories have one or two RCs in operation), suggesting these regions are in the early stages of RC implementation. Furthermore, through our working group, RECOLLECT International Research Consortium (https://www.researchintorecovery.com/recollect-international-research-consortium-rirc/), we have initiated the planning of RC implementations in new countries such as Brazil and Ukraine. These findings offer a roadmap for implementing and scaling RCs in such regions by identifying which aspects of RC operation require cultural adaptation and prioritised attention.

### Limitations

Four study limitations are noteworthy. First, alternative cross-cultural frameworks (eg, tightness-looseness, cultural values) could have been used, but data for many of the 28 countries and territories were unavailable, hindering meaningful comparisons. Critiques of Hofstede’s cultural model include treating nations as uniform cultural units and overlooking non-psychological cultural factors like socioeconomic and eco-social dynamics. Addressing these requires deeper, community-driven approaches, such as participatory research or culturally specific methods like Pagtatanong-tanong in the Philippines,^37^ which involves informal, conversational questioning within trusted relationships to elicit authentic and culturally grounded responses. Second, while the analysis included adjustments for key confounders, unmeasured confounders may still exist and impact the findings. Exclusion of RCs with incomplete data further limits robustness, and the uneven distribution of RCs across countries and territories constrains generalisability. Third, surveys completed by service managers may not fully capture the perspectives of other stakeholders, such as students. Although fully assessing all 12 components requires enough knowledge about the RC, future research should also incorporate student evaluations, addressing ethical considerations and ensuring rigorous sampling across contexts. Additionally, quantitative fidelity measures, like the RFM, may oversimplify complex RC characteristics,^38^ such as psychological safety, environmental impacts and relative importance of each component across cultures. Moreover, the reliance on a p value threshold of 0.05 for statistical significance may overlook nuances or potentially meaningful findings with higher p values, which could have added depth to the interpretation of these measures. Qualitative approaches could better capture these dimensions.^39^ Lastly, as cultures and practices evolve over time, ongoing research is needed to adapt and refine the cross-cultural understanding of RCs.

### Implications

This global study highlights the importance of tailoring RC operations to align with cultural norms, ensuring greater accessibility, equity and effectiveness worldwide. Two key implications emerge. First, the RFM can be further refined by incorporating underemphasised cultural characteristics into these identified items. This offers an opportunity to accelerate the global implementation of RCs by making the measure more culturally inclusive and adaptable. Second, adapting these seven operational elements to local cultural characteristics should be prioritised to optimise RC functionality and outcomes globally.

## Conclusions

This global study, encompassing RCs across 28 countries and territories, provides critical insights into the cultural influences shaping key fidelity components. It is one of the first to systematically examine how cultural characteristics impact RC operational elements and the RFM. The findings highlight that the key operational components—equality, learning, co-production, community focus, commitment to recovery, strengths-based practices and distinctive course offerings—can be adapted to enhance cultural inclusivity and effectiveness.

This research is novel in its global scope and its focus on cultural adaptation in RCs, addressing gaps in existing literature by offering specific, actionable guidance for fostering inclusivity and reducing mental health disparities. Future research should use qualitative methods to capture cultural nuances and engage diverse stakeholders to enhance adaptation processes. Regular evaluations will also be critical to sustain RC effectiveness as cultural contexts evolve. By applying these findings, RCs can expand their global impact, promoting recovery and addressing mental health inequalities in culturally diverse settings.

## Supplementary material

10.1136/gpsych-2024-102010 online supplemental file 1

10.1136/gpsych-2024-102010 online supplemental file 2

10.1136/gpsych-2024-102010 online supplemental file 3

## Data Availability

The data that support the findings of this study are available from the corresponding author on reasonable request. The data are not publicly available as they contain identifiable information about recovery colleges.
