# Supplementary material for: Cultural influences on fidelity components in recovery colleges: a study across 28 countries and territories
Source: Gen Psychiatr. 2025 May 27;38(3):e102010. doi: 10.1136/gpsych-2024-102010 (PMC12142041; doi:10.1136/gpsych-2024-102010)
Supplement: online supplemental file 2 [file gpsych-38-3-s002.docx]

**Supplemental Material 2: STROBE guidelines**

|  | **Item No.** | **Recommendation** | **Page No.** | **Relevant text from manuscript** |
| --- | --- | --- | --- | --- |
| **Title and abstract** | 1 | (a) Indicate the study’s design with a commonly used term in the title or the abstract | 1 | Cross-sectional study noted in Abstract. |
|  |  | (b) Provide in the abstract an informative and balanced summary of what was done and what was found | 1 | “Methods” reports what was done, and “Results” reports what was found in Abstract. |
| **Introduction** | | | |  |
| Background/rationale | 2 | Explain the scientific background and rationale for the investigation being reported | 4-6 | From "Recovery Colleges (RCs) are a relatively new mental health recovery support approach focused on education, skill development, and social support.”  To “Without this understanding, RC staff lack clear guidance on which aspects of their operations may need to be adapted and how to implement these changes effectively.” |
| Objectives | 3 | State specific objectives, including any prespecified hypotheses | 6 | "This study aimed to identify associations between Hofstede’s cultural dimension indices and each item of RFM in all RCs currently operating around the world.” |
| **Methods** | | | |  |
| Study design | 4 | Present key elements of study design early in the paper | 6 | "We conducted a cross-sectional, observational survey in two phases” |
| Setting | 5 | Describe the setting, locations, and relevant dates, including periods of recruitment, exposure, follow-up, and data collection | 6 | “All RCs, whose managers completed the RFM from August to October 2021 for the England survey, and from February to October 2022 for the international survey, were included. This study is a post-hoc analysis of data obtained from both the England ^20^ and international surveys,^2^ focusing on the cultural aspects of RCs and informed by previous hypothesis-generating cross-cultural analysis of the same dataset ^11 14 15 21^.” |
| Participants | 6 | (a) Cross-sectional study—Give the eligibility criteria, and the sources and methods of selection of participants | 6 | "*(1) Establishing RC inclusion criteria*  Since not all RCs identified as a “Recovery College” (e.g., “Recovery Academy”), we included any services that met three criteria, based on key RC components ^10^: (a) a focus on supporting personal recovery, (b) an emphasis on co-production, and (c) use of adult learning principles, all confirmed by service managers. Full details are reported elsewhere.^2^” |
| Variables | 7 | Clearly define all outcomes, exposures, predictors, potential confounders, and effect modifiers. Give diagnostic criteria, if applicable | 8-9 | Outcome variable, predictor variables, and confounder variables are defined. |
| Data sources/ measurement | 8 | For each variable of interest, give sources of data and details of methods of assessment (measurement). Describe comparability of assessment methods if there is more than one group | 8-9 | Outcome variables were all 12 items of the RECOLLECT Fidelity Measure. Predictor variables were four cultural characteristics of six obtained from Hofstede, based on the Value Survey Module 2013. Confounder variables were the percentage of GDP spent on health and the Gini coefficients. All sources are referenced. |
| Bias | 9 | Describe any efforts to address potential sources of bias | 16 | Potential sources of bias, such as uneven representation of countries, were acknowledged and addressed by excluding incomplete data. |
| Study size | 10 | Explain how the study size was arrived at | Figure 1 | Study size was determined by the number of RC managers completing the fidelity survey (n=169), representing 28 countries. |
| Quantitative variables | 11 | Explain how quantitative variables were handled in the analyses. If applicable, describe which groupings were chosen and why | 8-9 | The non-modifiable items of the RECOLLECT Fidelity Measure (RFM) were handled as ordinal variables, and the modifiable items were handled as categorical variables. Cultural Characteristics, and confounder variables were handled as continuous variables. |
| Statistical methods | 12 | (a) Describe all statistical methods, including those used to control for confounding | 9 | Mixed-effects ordinal logistic regression and, mixed-effects logistic regression models - efforts to control for confounding are described in the Statistical analysis section. |
|  |  | (b) Describe any methods used to examine subgroups and interactions | N/A | The examination of subgroups or interactions was not applicable in this study |
|  |  | (c) Explain how missing data were addressed | 9 | Complete case analysis - countries were omitted from analyses if data were missing. Imputation was not appropriate. |
|  |  | (d) Cross-sectional study—If applicable, describe analytical methods taking account of sampling strategy | N/A | A complex sampling strategy was not used in this study, so we did not need to adjust for this. |
|  |  | (e) Describe any sensitivity analyses | N/A | No sensitivity analyses were performed. |
| **Results** | | | | |
| Participants | 13 | (a) Report numbers of individuals at each stage of study—e.g. numbers potentially eligible, examined for eligibility, confirmed eligible, included in the study, completing follow-up, and analysed | 9 | The fidelity measure was completed by 169 (76%) RC managers from 28 countries, with over 55,000 students attending in total. |
|  |  | (b) Give reasons for non-participation at each stage | Figure 1 | Reasons for non-participation include ineligibility, non-contactable services, or inactive RCs. |
|  |  | (c) Consider use of a flow diagram | Figure 1 |  |
| Descriptive data | 14 | (a) Give characteristics of study participants (ego demographic, clinical, social) and information on exposures and potential confounders | Table 3 |  |
|  |  | (b) Indicate number of participants with missing data for each variable of interest | 9 | The statistical analysis section reports this (e.g., Gini coefficient missing for New Zealand = 2 RCs). |
|  |  | (c) Cohort study—Summarise follow-up time (e.g., average and total amount) | N/A |  |
| Outcome data | 15 | *Cross-sectional study—Report numbers of outcome events or summary measures* | Table 4 |  |
| Main results | 16 | (a) Give unadjusted estimates and, if applicable, confounder-adjusted estimates and their precision (ego, 95% confidence interval). Make clear which confounders were adjusted for and why they were included | Table 4 | Only adjusted associations were reported in order to focus on real-world applications, minimise potential misinterpretation, and maintain consistency with related studies. |
|  |  | (b) Report category boundaries when continuous variables were categorized | N/A | No continuous variables were categorised. |
|  |  | (c) If relevant, consider translating estimates of relative risk into absolute risk for a meaningful time period | N/A | We do not report relative risk. |
| Other analyses | 17 | Report other analyses done—e.g. analyses of subgroups and interactions, and sensitivity analyses | None |  |
| **Discussion** | | | | |
| Key results | 18 | Summarise key results with reference to study objectives | 13 | "This study investigated associations between Hofstede’s cultural dimensions and each item of the RECOLLECT Fidelity Measure in all RCs operating in the world. The results revealed notable links between cultural characteristics and both non-modifiable and modifiable fidelity items. Of the 12 items, seven were associated with at least one cultural characteristic. These findings underscore the substantial cultural influences shaping RC operations.” |
| Limitations | 19 | Discuss limitations of the study, taking into account sources of potential bias or imprecision. Discuss both direction and magnitude of any potential bias | 16 | A paragraph starting with "Four study limitations are noteworthy.” |
| Interpretation | 20 | Give a cautious overall interpretation of results considering objectives, limitations, multiplicity of analyses, results from similar studies, and other relevant evidence | 14-16 | From "The association between Short-Term Orientation and higher Equality scores reflects the emphasis on practical and egalitarian approaches in Short-Term-Oriented cultures”   To the end of the limitation section: “…ongoing research is needed to adapt and refine cross-cultural understanding of RCs.” |
| Generalisability | 21 | Discuss the generalisability (external validity) of the study results | 14-15 | From " The association between Short-Term Orientation and higher Equality scores reflects the emphasis on practical and egalitarian approaches in Short-Term-Oriented cultures”  To “These findings offer a roadmap for implementing and scaling RCs in such regions. |
| **Other information** | |  | | |
| Funding | 22 | Give the source of funding and the role of the funders for the present study and, if applicable, for the original study on which the present article is based | 6 | "This study is part of the RECOLLECT 2 programme, a five-year (2020-2025) National Institute for Health and Care Research (NIHR)-funded project investigating the effectiveness and cost-effectiveness of RCs.” |
