# Supplementary material for: Cultural influences on fidelity components in recovery colleges: a study across 28 countries and territories
Source: Gen Psychiatr. 2025 May 27;38(3):e102010. doi: 10.1136/gpsych-2024-102010 (PMC12142041; doi:10.1136/gpsych-2024-102010)
Supplement: online supplemental file 3 [file gpsych-38-3-s003.docx]

**Supplemental Material 3: Fidelity scores for each item per country**

|  | | **Non-modifiable items** | | | | | | |  |
| --- | --- | --- | --- | --- | --- | --- | --- | --- | --- |
| **Country (n=28)** | **Recovery College (n=168/220: responded/total)** | **(1) Equality** | **(2) Learning** | **(3) Tailored to Students** | **(4) Co-production** | **(5) Social Connectedness** | **(6) Community Focus** | **(7) Commitment to Recovery** | **Total** |
|  |  | *Median (IQR)* | *Median (IQR)* | *Median (IQR)* | *Median (IQR)* | *Median (IQR)* | *Median (IQR)* | *Median (IQR)* | *Median (IQR)* |
| *Africa (n=1)* | *2/2* |  |  |  |  |  |  |  |  |
| Uganda | 2/2 | Blinded | Blinded | Blinded | Blinded | Blinded | Blinded | Blinded | Blinded |
| *Asia (n=3)* | *13/15* |  |  |  |  |  |  |  |  |
| Hong Kong | 2/2 | Blinded | Blinded | Blinded | Blinded | Blinded | Blinded | Blinded | Blinded |
| Japan | 9/11 | 2 (1 to 2) | 1 (1 to 1) | 1 (1 to 1) | 1 (0 to 1) | 1 (1 to 2) | 1 (1 to 1) | 1 (1 to 2) | 7 (6 to 10) |
| Thailand | 2/2 | Blinded | Blinded | Blinded | Blinded | Blinded | Blinded | Blinded | Blinded |
| *Europe (n=20)* | *128/169* |  |  |  |  |  |  |  |  |
| Belgium | 10/14 | 1 (0 to 2) | 1 (1 to 1) | 1 (1 to 1) | 2 (1 to 2) | 1 (1 to 1) | 1 (1 to 2) | 2 (1 to 2) | 8.5 (8 to 10) |
| Bulgaria | 1/1 | Blinded | Blinded | Blinded | Blinded | Blinded | Blinded | Blinded | Blinded |
| Czechia | 1/1 | Blinded | Blinded | Blinded | Blinded | Blinded | Blinded | Blinded | Blinded |
| Denmark | 9/9 | 1 (1 to 2) | 1 (1 to 1) | 1 (1 to 1) | 1 (0 to 2) | 2 (1 to 2) | 1 (0 to 2) | 2 (1 to 2) | 8 (6 to 9) |
| England | 63/88 | 2 (2 to 2) | 1 (1 to 2) | 2 (1 to 2) | 2 (1 to 2) | 1 (1 to 2) | 2 (1to 2) | 2 (1 to 2) | 11 (9 to 13) |
| Estonia | 2/2 | Blinded | Blinded | Blinded | Blinded | Blinded | Blinded | Blinded | Blinded |
| Finland | 2/2 | Blinded | Blinded | Blinded | Blinded | Blinded | Blinded | Blinded | Blinded |
| France | 1/1 | Blinded | Blinded | Blinded | Blinded | Blinded | Blinded | Blinded | Blinded |
| Germany | 3/3 | 2 (2 to 2) | 1 (0 to 2) | 1 (1 to 1) | 1 (0 to 1) | 1 (1 to 2) | 1 (0 to 1) | 2 (1 to 2) | 9 (6 to 10) |
| Hungary | 2/3 | Blinded | Blinded | Blinded | Blinded | Blinded | Blinded | Blinded | Blinded |
| Iceland | 1/1 | Blinded | Blinded | Blinded | Blinded | Blinded | Blinded | Blinded | Blinded |
| Ireland | 7/11 | 2 (2 to 2) | 2 (1 to 2) | 1 (1 to 2) | 2 (1 to 2) | 2 (1 to 2) | 2 (1 to 2) | 2 (1 to 2) | 11 (10 to 13) |
| Italy | 4/4 | 2 (1 to 2) | 1.5 (1 to 2) | 1 (0.5 to 1.5) | 1 (0 to 2) | 1 (1 to 1) | 1.5 (0.5 to 2) | 0.5 (0 to 1) | 7.5 (5 to 10.5) |
| Netherlands | 2/2 | Blinded | Blinded | Blinded | Blinded | Blinded | Blinded | Blinded | Blinded |
| Northern Ireland | 3/4 | 2 (1 to 2) | 2 (1 to 2) | 2 (2 to 2) | 2 (2 to 2) | 2 (1 to 2) | 1 (1 to 2) | 2 (2 to 2) | 13 (10 to 14) |
| Norway | 4/5 | 2 (2 to 2) | 1.5 (0.5 to 2) | 2 (2 to 2) | 2 (2 to 2) | 2 (1.5 to 2) | 1 (1 to 1) | 2 (2 to 2) | 12.5 (11 to 13) |
| Scotland | 3/3 | 2 (2 to 2) | 1 (0 to 1) | 2 (2 to 2) | 1 (1 to 1) | 1 (1 to 2) | 2 (2 to 2) | 2 (1 to 2) | 11 (9 to 12) |
| Spain | 3/6 | 1 (0 to 2) | 1 (0 to 1) | 2 (1 to 2) | 0 (0 to 2) | 1 (1 to 1) | 1 (1 to 1) | 1 (1 to 1) | 6 (5 to 10) |
| Sweden | 3/3 | 2 (1 to 2) | 2 (1 to 2) | 1 (1 to 2) | 1 (1 to 2) | 2 (0 to 2) | 1 (1 to 2) | 2 (1 to 2) | 11 (6 to 14) |
| Switzerland | 3/4 | 2 (1 to 2) | 1 (1 to 1) | 1 (1 to 1) | 1 (0 to 2) | 1 (1 to 1) | 0 (0 to 1) | 1 (1 to 2) | 8 (5 to 9) |
| Wales | 1/2 | Blinded | Blinded | Blinded | Blinded | Blinded | Blinded | Blinded | Blinded |
| *Oceania (n=2)* | *9/11* |  |  |  |  |  |  |  |  |
| Australia | 7/9 | 2 (2 to 2) | 1 (1 to 2) | 1 (1 to 2) | 2 (1 to 2) | 1 (1 to 2) | 1 (0 to 2) | 2 (1 to 2) | 10 (6 to 13) |
| New Zealand | 2/2 | Blinded | Blinded | Blinded | Blinded | Blinded | Blinded | Blinded | Blinded |
| *North America (n=1)* | *16/23* |  |  |  |  |  |  |  |  |
| Canada | 16/23 | 2 (2 to 2) | 1 (1 to 2) | 1 (1 to 2) | 2 (1 to 2) | 1 (1 to 2) | 1 (1 to 2) | 2 (2 to 2) | 10.5 (9 to 12) |

Countries with data from <3 Recovery Colleges were blinded for anonymity purposes.

|  |  | **Modifiable items** | | | | |
| --- | --- | --- | --- | --- | --- | --- |
| **Country (n=28)** | **Recovery College (n=168/220: responded/total)** | **(8) Available to All** | **(9) Location** | **(10) Distinctiveness of Course Content** | **(11) Strengths-based** | **(12) Progressive** |
|  |  | *Type 1 N(%)* | *Type 1 N(%)* | *Type 1 N(%)* | *Type 1 N(%)* | *Type 1 N(%)* |
| *Africa (n=1)* | *2/2* |  |  |  |  |  |
| Uganda | 2/2 | Blinded | Blinded | Blinded | Blinded | Blinded |
| *Asia (n=3)* | *13/15* |  |  |  |  |  |
| Hong Kong | 2/2 | Blinded | Blinded | Blinded | Blinded | Blinded |
| Japan | 9/11 | 8 (88.9) | 7 (77.8) | 6 (66.7) | 7 (77.8) | 7 (77.8) |
| Thailand | 2/2 | Blinded | Blinded | Blinded | Blinded | Blinded |
| *Europe (n=21)* | *128/169* |  |  |  |  |  |
| Belgium | 10/14 | 9 (90.0) | 6 (60.0) | 6 (60.0) | 0 (0.0) | 9 (90.0) |
| Bulgaria | 1/1 | Blinded | Blinded | Blinded | Blinded | Blinded |
| Czechia | 1/1 | Blinded | Blinded | Blinded | Blinded | Blinded |
| Denmark | 9/9 | 7 (77.8) | 2 (22.2) | 2 (22.2) | 2 (22.2) | 8 (88.9) |
| England | 63/88 | 44 (69.8) | 30 (47.6) | 27 (42.9) | 13 (20.6) | 41 (65.1) |
| Estonia | 2/2 | Blinded | Blinded | Blinded | Blinded | Blinded |
| Finland | 2/2 | Blinded | Blinded | Blinded | Blinded | Blinded |
| France | 1/1 | Blinded | Blinded | Blinded | Blinded | Blinded |
| Germany | 3/3 | 3 (100.0) | 1 (33.3) | 3 (100.0) | 1 (33.3) | 3 (100.0) |
| Hungary | 2/3 | Blinded | Blinded | Blinded | Blinded | Blinded |
| Iceland | 1/1 | Blinded | Blinded | Blinded | Blinded | Blinded |
| Ireland | 7/11 | 5 (71.4) | 3 (42.9) | 2 (28.6) | 2 (28.6) | 6 (85.7) |
| Italy | 4/4 | 3 (75.0) | 2 (50.0) | 0 (0.0) | 2 (50.0) | 2 (50.0) |
| Netherlands | 2/2 | Blinded | Blinded | Blinded | Blinded | Blinded |
| Northern Ireland | 3/4 | 3 (100.0) | 1 (33.3) | 3 (100.0) | 0 (0.0) | 2 (66.7) |
| Norway | 4/5 | 4 (100.0) | 3 (75.0) | 2 (50.0) | 0 (0.0) | 2 (50.0) |
| Scotland | 3/3 | 3 (100.0) | 3 (100.0) | 1 (33.3) | 0 (0.0) | 2 (66.7) |
| Spain | 3/6 | 1 (33.3) | 0 (0.0) | 1 (33.3) | 1 (33.3) | 1 (33.3) |
| Sweden | 3/3 | 0 (0.0) | 1 (33.3) | 0 (0.0) | 1 (33.3) | 1 (33.3) |
| Switzerland | 3/4 | 3 (100.0) | 1 (33.3) | 1 (33.3) | 1 (33.3) | 3 (100.0) |
| Wales | 1/2 | Blinded | Blinded | Blinded | Blinded | Blinded |
| *Oceania (n=2)* | *9/11* |  |  |  |  |  |
| Australia | 7/9 | 4 (57.1) | 3 (42.9) | 4 (57.1) | 3 (42.9) | 5 (71,4) |
| New Zealand | 2/2 | Blinded | Blinded | Blinded | Blinded | Blinded |
| *North America (n=1)* | *16/23* |  |  |  |  |  |
| Canada | 16/23 | 11 (68.7) | 9 (56.2) | 10 (62.5) | 4 (25.0) | 12 (75.0) |

Countries with data from <3 Recovery Colleges were blinded for anonymity purposes.
